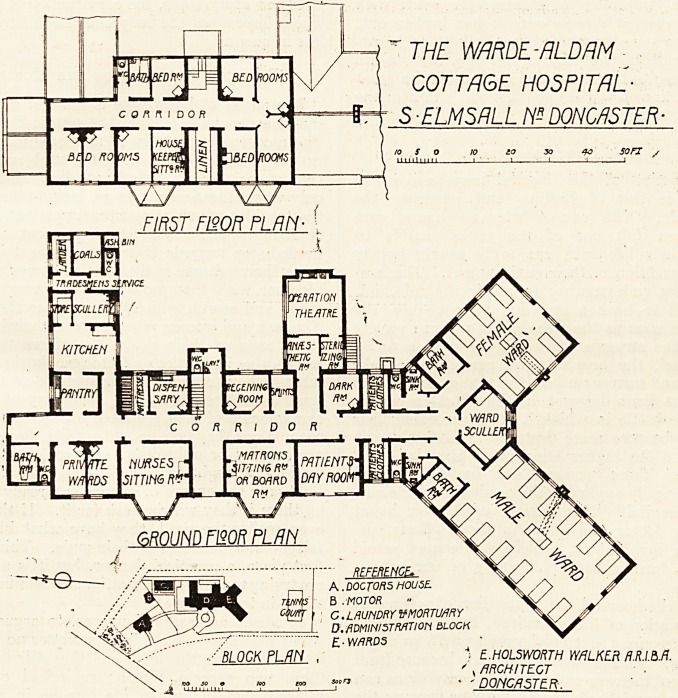# Hospital Architecture and Construction

**Published:** 1911-10-07

**Authors:** 


					October 7, 1911. THE HOSPITAL 21
HOSPITAL ARCHITECTURE AND CONSTRUCTION.
Communications on this subject should be marked "Architecture'' in the left-hand top corner of the envelope.
Warde-Aldam Cottage Hospital.
This is being erected at Frickley for the Carlton Main
Colliery Company, from the design by Mr. E. Holsworth
Walker, A.R.I.B.A., of Doncaster.
Reference to the block plan shows that the scheme con-
sists of male and female wards, administrative block,
laundry block, with mortuary and post-mox-tem room ad-
joining, and medical officer's residence.
The hospital is built on the Y-shaped plan, the two arms
of the Y forming the male and female wards respectively,
with the Duty Room placed between, and commanding a
view of both wards.
Accommodation is provided for twelve males and six
females. The sanitary arrangements in each case are
identical, consisting of bath-room, sink-room, and w.c.
The original design provided for sanitary towers cut off
by cross-ventilation, but had to be omitted on the score
of expense. The wards are heated by central Shoreland
stoves.
Patients' clothes stores are provided, cut off from the
administration block by swing-doors. The floors through-
out are of " Terralith," except the ward kitchen, operat-
ing, anaesthetic, and sterilising rooms, which have Terrazzo
marble.
On the east front is built the operating-room and its
adjuncts. The operating-room has a good window and top-
light, facing north, and is ventilated by a ceiling extract-
fan.
The administrative portion provides nurses' and matron's-
r.itting-rooms, receiving-room, dispensary, and several use-
ful stores.
At the north end of the administration department is the
kitchen and offices with two private wards on the west
front, to which latter is attached a bath-room and w.c.
The first floor of the administrative block provides eight:
bedrooms, housekeeper's sitting-room, and the usual offices.
The whole of the several buildings are built with local
pressed bricks, the first floor being stuccoed. The roofs are-
covered with hand-made brindle tiles.
" THE WRDL-RLDRM
COTTAGE HOSPITAL-
S-ELMSRLL W DONCASTER-
10 30 40 son
FJBST FtSOR PLSTT '
?>
ttutvm
CORRIDOR
WARD
IXULLL
PRl\foTE I NURSES r h??^ I MTIENTfi || kcLT^ V>?
W/faDS \5ITTIN6R>r ^0R BOARD \DAY v \S
/?f , <y
<9 %
GROUND F120R PLAN "V?/v ^y^/K
-,- ; ;  REFERENCE. V//
A. DOCTORS HOUSE. ^
B . MOTOR
C. LAUNDRY V MORTUARY
D. ADMINISTRATION BLOCK
E-WARDS
) E.HOLSWORTH WALKER A.Rib.ft.
' , ARCHITECT
' - DONCASTER

				

## Figures and Tables

**Figure f1:**